# A genetic switch involving EGR1 and EZH2 links aging and development across human tissues

**DOI:** 10.64898/2026.07.29.741427

**Published:** 2026-07-30

**Authors:** Neha Khetan, Jiashun Zheng, Changhui Deng, Evan Wu, Hao Li

## Abstract

Aging is often regarded as the last chapter of development. There is growing evidence that aging and development are mechanistically linked. Here we describe a genetic switch involving EZH2 and EGR1 as opposing regulatory nodes that appears to connect aging and development across human tissues. We show that this switch defines two distinct states in fibroblasts: a proliferative (EZH2-high/EGR1-low) state and a non-proliferative and extracellular matrix-expressing (EGR1-high/EZH2-low) state. During replicative aging, cells shift from the proliferative state to the non-proliferative state and targeting either regulatory node—by overexpressing EZH2 or suppressing EGR1—rejuvenates aged fibroblasts. We provide evidence that this switch is involved in the transition from neural progenitor cells to matured neurons during brain development and becomes destabilized during aging, partially reversing the gene expression program established during development. We observed that this same switch becomes blurred and biased towards EGR1-high/EZH2-low state during the aging of muscle stem cells and hematopoietic stem cells. Since genetic switches are widely used in development to establish and reinforce cellular identity, we propose that attenuation of developmental switches may underlie aging across diverse organs and tissues, and partially accounts for the erosion of the epigenetic landscape and the loss of cellular identity.

## Full Text Availability

The license terms selected by the author(s) for this preprint version do not permit archiving in PMC. The full text is available from the preprint server.

